# Population Structure and Evolution after Speciation of the Hokkaido Salamander (*Hynobius retardatus*)

**DOI:** 10.1371/journal.pone.0156815

**Published:** 2016-06-03

**Authors:** Masatoshi Matsunami, Takeshi Igawa, Hirofumi Michimae, Toru Miura, Kinya Nishimura

**Affiliations:** 1 Laboratory of Ecological Genetics, Graduate School of Environmental Science, Hokkaido University, Sapporo, 060–0810, Japan; 2 Graduate School of International Development and Cooperation, Hiroshima University, Higashi-Hiroshima, 739–8526, Japan; 3 School of Pharmacy, Department of Clinical Medicine (Biostatistics), Kitasato University, Tokyo, 108–8641, Japan; 4 Graduate School of Fisheries Sciences, Hokkaido University, Hakodate, 041–8611, Japan; University of Innsbruck, AUSTRIA

## Abstract

The Hokkaido salamander (*Hynobius retardatus*) is endemic to Hokkaido Island, Japan, and shows intriguing flexible phenotypic plasticity and regional morphological diversity. However, to date, allozymes and partial mitochondria DNA sequences have provided only an outline of its demographic histories and the pattern of its genetic diversification. To understand the finer details of the population structure of this species and its evolution since speciation, we genotyped five regional populations by using 12 recently developed microsatellite polymorphic markers. We found a clear population structure with low gene flow among the five populations, but a close genetic relationship between the Teshio and Kitami populations. Our demographic analysis suggested that Teshio and Erimo had the largest effective population sizes among the five populations. These findings regarding the population structure and demography of *H*. *retardatus* improve our understanding of the faunal phylogeography on Hokkaido Island and also provide fundamental genetic information that will be useful for future studies.

## Introduction

One of the major goals of molecular ecology is to understand the current genetic structure and pattern of genetic diversity of organisms. Moreover, these data are useful for conservation management, because genetic diversity is an essential factor in morphological and behavioral phenotypic variation. Salamanders have been used as model species for studies of genetic structure and diversity [1] because gene flow among populations is restricted by their low dispersal ability [2].

The salamander family Hynobiidae comprises over 50 species distributed entirely within Asia. Extensive investigations of the phylogenetic relationship among species of this family using mitochondrial DNA [3–5] and nuclear genes [6] have shown with high confidence that genus *Hynobius* is monophyletic in East Asia. Most salamander species belonging to this genus are endemic to the Japanese archipelago, and phylogenetic and phylogeographic analyses of local populations of several *Hynobius* species, including *H*. *boulengeri* [7], *H*. *kimurae* [8], *H*. *naevius* [9], *H*. *nebulosus* [10], *H*. *tokyoensis* [11, 12], *H*. *yatsui* [13], and *H*. *dunni* [14], have been conducted in recent decades.

Another *Hynobius* species, the Hokkaido salamander (*H*. *retardatus*) is endemic to Hokkaido Island, the northernmost island of the Japanese archipelago. This species has a number of interesting characteristics, including neoteny [15], temperature-dependent sex differentiation [16], and predator- or prey-induced phenotypic plasticity [17–21]. Furthermore, the reported diversification of several life-history traits among regional populations [22] has motivated phylogenetic and phylogeographic studies based on intensive genetic analyses.

Efforts have begun to elucidate genetic diversity in this species. Matsui et al. [23] showed by allozyme electrophoresis of three *H*. *retardatus* populations that their genetic differences did not correlate with morphological differences but with geographic location. In addition, Azuma et al. [24] recently identified three mitochondrial haplotype groups, each with a different geographic distribution. However, current genetic structure among local populations and the evolutionary history of those populations remain obscure owing to an overall paucity of genetic analysis data.

Insight into the biogeographic history of Hokkaido Island has been provided by phylogeographic studies of the Hokkaido salamander. The divergence of the focal species from other salamanders is estimated to have occurred 14–18 million years ago (Ma) [6]. Because this speciation time approximately corresponds to the emergence of the Japanese archipelago during the Miocene, Pliocene, and Pleistocene epochs, this species has experienced unstable geological and climate conditions in its current habitat. Hokkaido Island remained connected to Sakhalin Island and the Eurasian Continent by land bridges until the end of the last glacial period, about 10,000 years ago [25]. Most vertebrates in Hokkaido migrated from continental Eurasia via these land bridges. For example, the dark red-backed vole (*Myodes rex*), which is endemic to Hokkaido Island, immigrated into Hokkaido from the north by the Middle Pleistocene [26], and the brown bear (*Ursus arctos*) may have migrated to Hokkaido on at least three separate occasions before the disappearance of the land bridges [27]. Another example is the wood mouse (*Apodemus speciosus*), which colonized Hokkaido Island when the sea level dropped during glacial periods of the Quaternary [28]. Unlike the potential habitable area of these terrestrial organisms, the habitable area of the Hokkaido salamander is restricted to lentic freshwater environments; as a result, the mobile ability of the species is low, a characteristic that it shares with other species of freshwater biota. Therefore, by studying the phylogeography of this species, insight can gained into the biogeographic history of Hokkaido lentic freshwater biota.

In this study, we examined gene flow and the population structure of the Hokkaido salamander by using recently developed polymorphic microsatellite markers. We found low gene flow between populations and a distinct genetic structure. We expected the genetic information obtained by this study to improve our understanding of the phylogeography of this salamander and the overarching biogeography of the lentic freshwater biota on Hokkaido Island.

## Materials and Methods

### Sample collection

**Fig 1:**
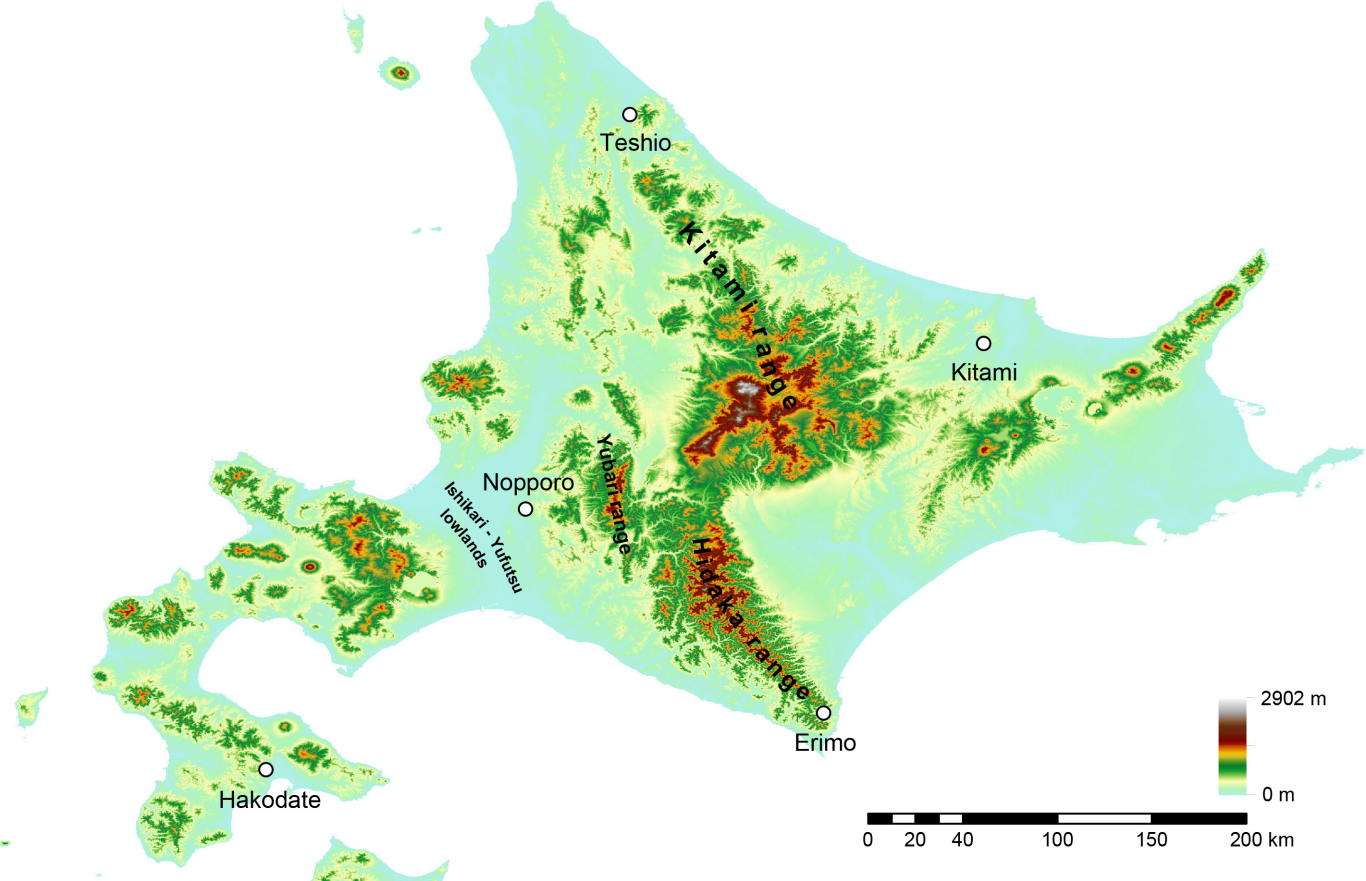
Sampling location of Hokkaido salamanders. Map showing the locality where each population was sampled. The localities are characterized by different mitochondrial DNA haplotype groups. Topographic map was projected by using ArcGIS 9.3 based on a 10-m grid digital elevation map provided by The Geospatial Information Authority of Japan.

We collected *H*. *retardatus* eggs in the breeding season (April to May) each year during 2013–2015 from ponds of five local populations (Fig 1): Erimo (42°07’N, 143°15’E), Nopporo (43°06’N, 141°50’E), Hakodate (41°88’N, 140°57’E), Teshio (45°04’N, 142°00’E), and Kitami (43°53’N, 144°00’). At each locality, 10–20 egg clutches were collected. To gain sufficient volume of DNA, we cultivated these salamanders’ eggs in the laboratory. Each salamander egg cluster was placed into a different stock tank (33.4 cm × 20 cm × 10 cm high) filled with 2 L of aged tap water and kept in the laboratory at 4°C. After the eggs hatched, we randomly selected hatchlings for genotyping to avoid choosing ones with the same parents. Genomic DNA for genotyping was extracted from tail-tip tissues of approximately 20 individuals from each locality with a DNA suisui-F kit (Rizo, Tsukuba, Japan) following the manufacturer’s instructions.

### Ethic statement

The research reported herein was approved by Institutional Animal Care and Use Committee of National University Corporation Hokkaido University. Because subjects of regulation by the committee are mammalians, birds, and reptiles, approval number was not issue for our experiments. The focal species was not enlisted as endangered and protected species in any districts in their distribution area at present, and thus no permission was required for the procedure of this study. All salamanders used in this study were deeply euthanized with 0.05% benzocaine in fresh water and stored individually in ethanol, and used for DNA extraction. This was carried out in accordance with the recommendations in the Hokkaido University Manual for Implementing Animal Experimentation.

### Genotyping

We genotyped 12 microsatellite loci of *H*. *retardatus* following to the method described by Matsunami et al. [29]. From the resultant genotypes of individuals, we calculated observed and expected heterozygosities (*H*_O_ and *H*_E_), the number of alleles (*N*_A_), and the inbreeding coefficient (*F*_IS_) with GENALEX 6.5 software [30]. We performed tests for deviation from Hardy-Weinberg equilibrium (HWE) and linkage disequilibrium (LD) with Genepop ver. 4.2 software [31–32]. Existence of null alleles often leads to overestimation of *F*_ST_ [33–34]. We used MICRO-CHECKER V2.2.3 [35] to check for the presence of null alleles.

### Population structure analysis

To infer the phylogenetic relationships among the five populations, pairwise genetic distance and a phylogenetic tree based on the *D*_A_ distance of Nei et al. [36] with 1000 bootstrap iterations were calculated with POPTREE2 software [37]. Genetic population structures were analyzed with STRUCTURE version 2.3.2 [38], which uses a Bayesian clustering method to assign individuals to genetic population units. To explore the genetic structure of each population, we conducted multiple analyses while varying the number of Bayesian clusters (*K*) from 2 to 9. We also used admixture models for Markov Chain Monte Carlo (MCMC) inference with prior information on the locality of samples (LOCPRIOR) and correlated allele frequency. We ran 1,000,000 MCMC repetitions after discarding the first 100,000 iterations as burn-in, and took 10 repeated simulations for each *K* estimation. To estimate the optimal *K* value, we analyzed our results according to the method of Evanno et al. [39], which is implemented in the STRUCTURE HARVESTER web tool [40]. In this method, log-likelihood values of the 10 repeated runs for each *K* value and their variances are used to calculate Δ*K*. The average of each replicate cluster analysis was calculated by CLUMPP version 1.1.2 software [41]. The results of the calculations were visualized with DISTRUCT version 1.1 software [42].

To infer subgroups among the five local populations, we estimated the effective population size of each group and the migration rate among the groups. We used two programs to estimate gene flow: BAYESASS+ version 1.2 [43] estimates gene flow by a genetic assignment method, and MIGRATE version 3.6.11 [44] estimates gene flow and effective population sizes by a coalescent method. Of relevance here is that the genetic assignment method adopted by the BAYESASS+ typically measures recent migration rates, whereas the coalescent method adopted by MIGRATE estimates long-term migration rates [45]. BAYESASS+ uses fully Bayesian MCMC resampling to estimate the migration rate (*m*), allele frequency (*P*), and inbreeding value (*F*) as variable. We ran a total of 3,000,000 MCMC iterations and sampled the chain every 2000 iterations, discarding the first 1,000,000 iterations as burn-in. MIGRATE uses coalescent simulations to estimate parameter *M*, which is proportional to the pairwise migration rate (*M* = *m*/*μ*, where *m* = migration rate per generation and, *μ* = mutation rate per generation) and parameter Θ, which is proportional to the effective population size *N*_e_ (Θ = 4*N*_e_μ) of each population. We ran 10 short chains with 50,000 generations, sampled every 20 generations, and 8 long chains with 5000 generations, sampled every 20 generations.

## Results

### Genotyping

We genotyped a total of 103 individuals from five local populations using the 12 microsatellite loci (Table 1; S1 Table). The total number of alleles ranged from 3 to 20 with a mean of 8.667 per locus (S2 Table). An *Hret02* allele was fixed in Nopporo population, and alleles of *Hret05*, *Hret06*, *Hret07*, and *Hret08* were fixed in the Hakodate population. Microchecker program indicated null alleles in five loci (*Hret01*, *Hret02*, *Hret03*, *Hret07*, and *Hret09*) in some populations. Observed heterozygosity ranged from 0.208 to 0.664 with a mean of 0.440 per locus. We conducted 54 HWE tests representing every polymorphic locus/population combination (α = 0.00019 after Bonferroni correction) and detected significant deviation from HWE in four tests of four loci (*Hret01* in Teshio, *Hret03* in Kitami, *Hret07* in Teshio, and *Hret09* in Teshio). Because none of these loci showed significant HWE deviation in the other populations, we did not exclude them from the analysis. We also conducted 279 LD tests representing all pairwise comparisons of loci in each population, and detected no significant deviation of LD (α = 0.000036 after Bonferroni correction). To confirm the accuracy and reproducibility of our genotyping, we genotyped randomly selected samples twice; in each population, we found only minor differences in the number of alleles (*N*_A_), observed heterozygosity (*H*_O_), expected heterozygosity (*H*_E_), and inbreeding coefficient (*F*_IS_). We subsequently conducted analyses based on loci having no null alleles. Then, we confirmed that the results were qualitatively similar.

**Table 1:** Genotypic data of 12 microsatellite loci in populations of the Hokkaido salamander.

Population	*N*	*N*_A_	*H*_O_	*H*_E_	*F*_IS_
**Erimo**	20	4.167	0.590	0.585	0.001
**Nopporo**	20	3.750	0.504	0.451	-0.106
**Hakodate**	20	2.667	0.294	0.294	0.007
**Teshio**	21	4.000	0.348	0.485	0.225
**Kitami**	22	3.667	0.466	0.491	0.019

Abbreviations: *F*_IS_, inbreeding coefficient; *H*_O_, observed heterozygosity; *H*_E_, expected heterozygosity; *N*, number of individuals; *N*_A_, number of alleles

### Population structure analysis

**Fig 2:**
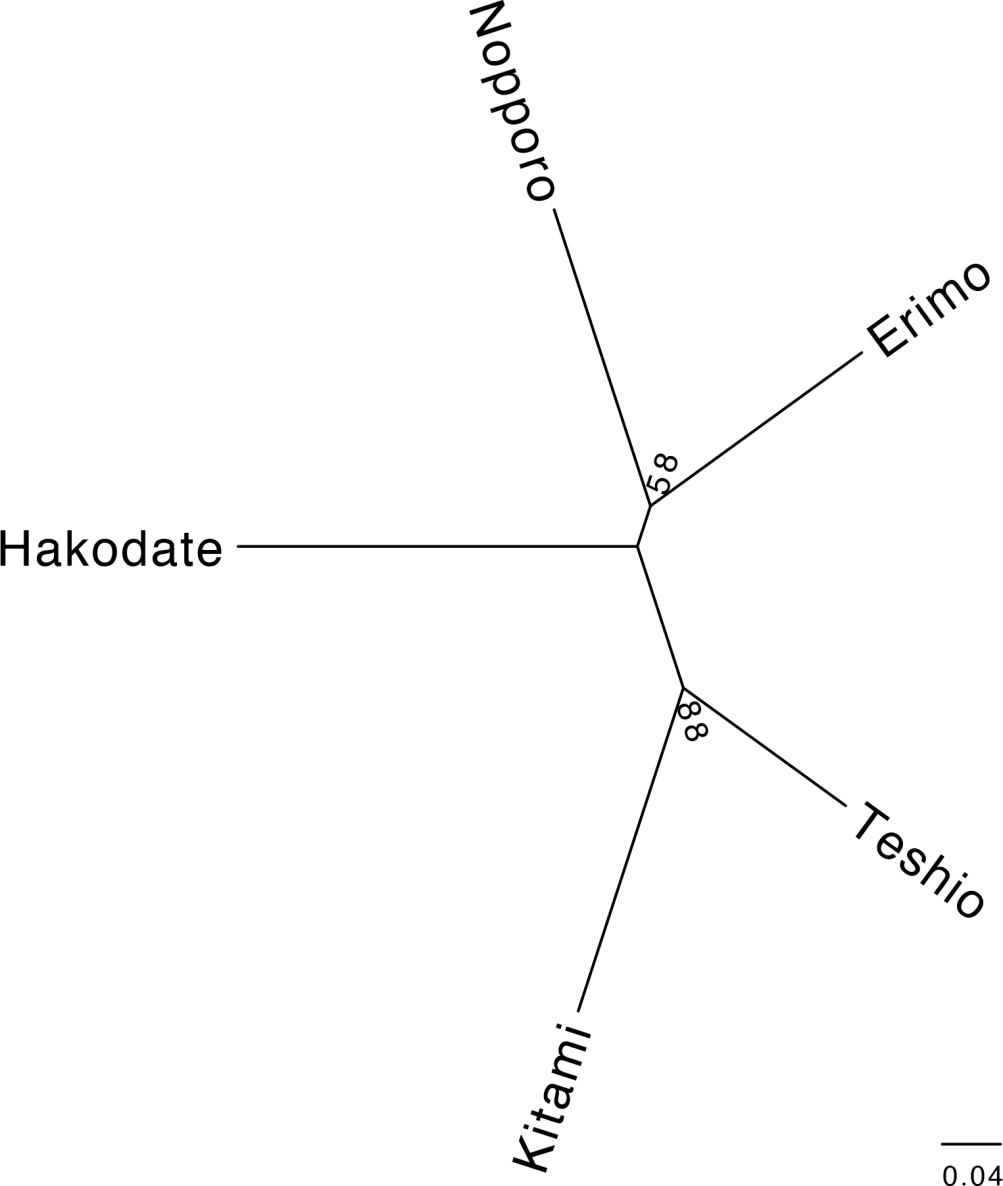
Phylogenetic relationships among five local populations of Hokkaido salamanders. This neighbor-joining phylogenetic tree was reconstructed by using Nei’s *D*_A_ distances. The number at each node indicates the bootstrap probability (%) with 1000 replications.

Pairwise *F*_ST_ and Nei’s *D*_A_ distances among populations ranged from 0.162 to 0.348 and from 0.613 to 1.166, respectively (S3 Table). The pairwise *F*_ST_ and Nei’s *D*_A_ distances between Kitami and Hakodate were largest, suggesting that these populations are highly diverged, whereas Nei’s *D*_A_ distances between Kitami and Teshio were smallest. A neighbor-joining phylogenetic tree was reconstructed with these Nei’s *D*_A_ distances (Fig 2) shows the Kitami and Teshio populations clustered with a high bootstrap probability (88%); the Nopporo and Erimo populations are also clustered, but the statistical support is low (58%).

**Fig 3:**
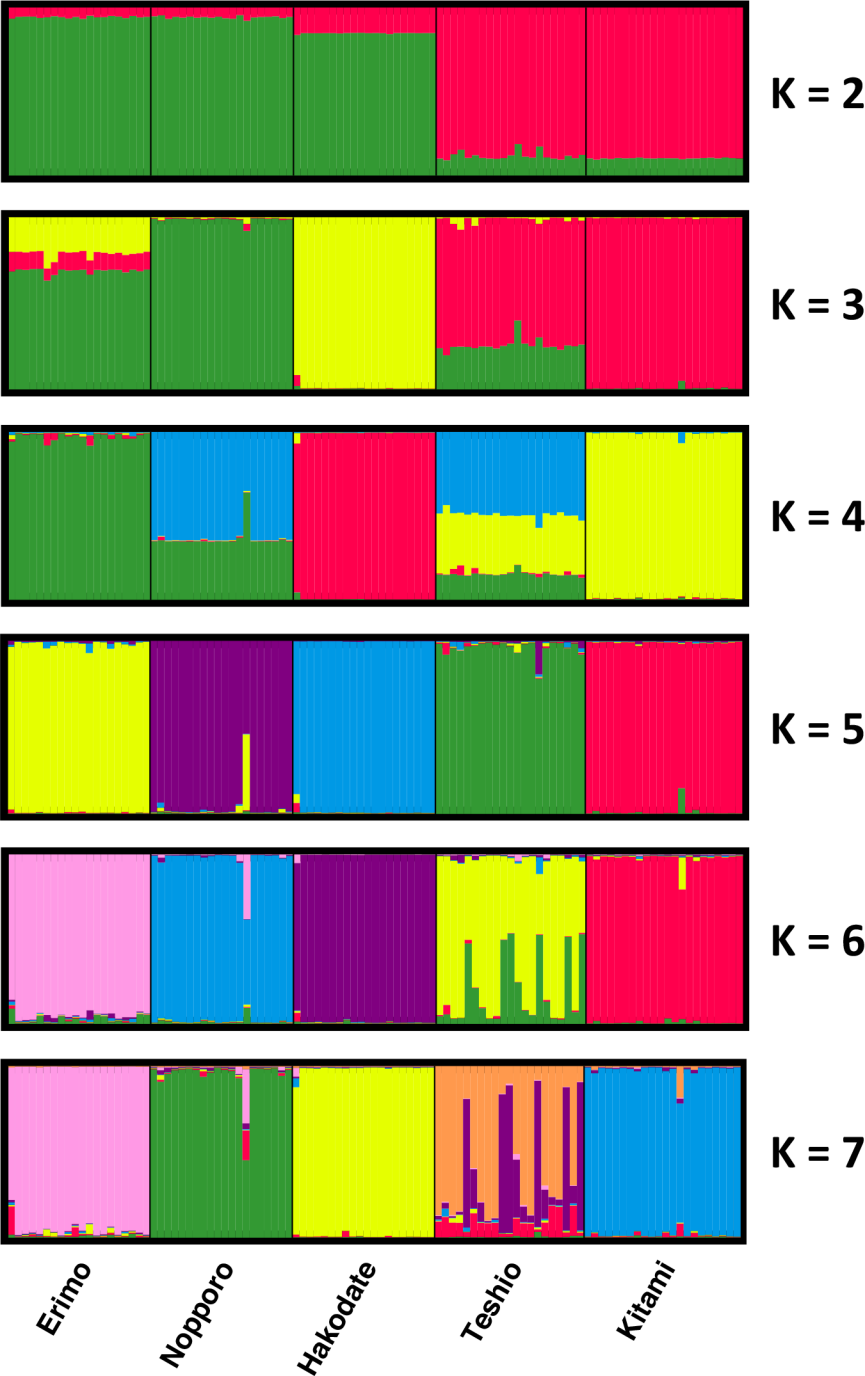
Assigned genetic clusters of the five local populations. Structure analysis was conducted for different *K* values (*K* = 2–6). The population structure was optimal (based on Δ*K*) for *K* = 5.

We also inferred subgroupings of the five populations with distinctive multi-locus allele frequencies by calculating Δ*K* from *K* = 2 to *K* = 9 with the STRUCTURE program (Fig 3). In these calculations, Δ*K* supports that *K* = 5 as the most suitable population structure model, indicating that each population is genetically distinct. In addition, the populations were clearly divided into two major groups (Erimo–Nopporo–Hakodate and Teshio–Kitami) at *K* = 2; thus the population structure analysis result is consistent with the phylogenetic tree reconstruction (Fig 2).

**Fig 4:**
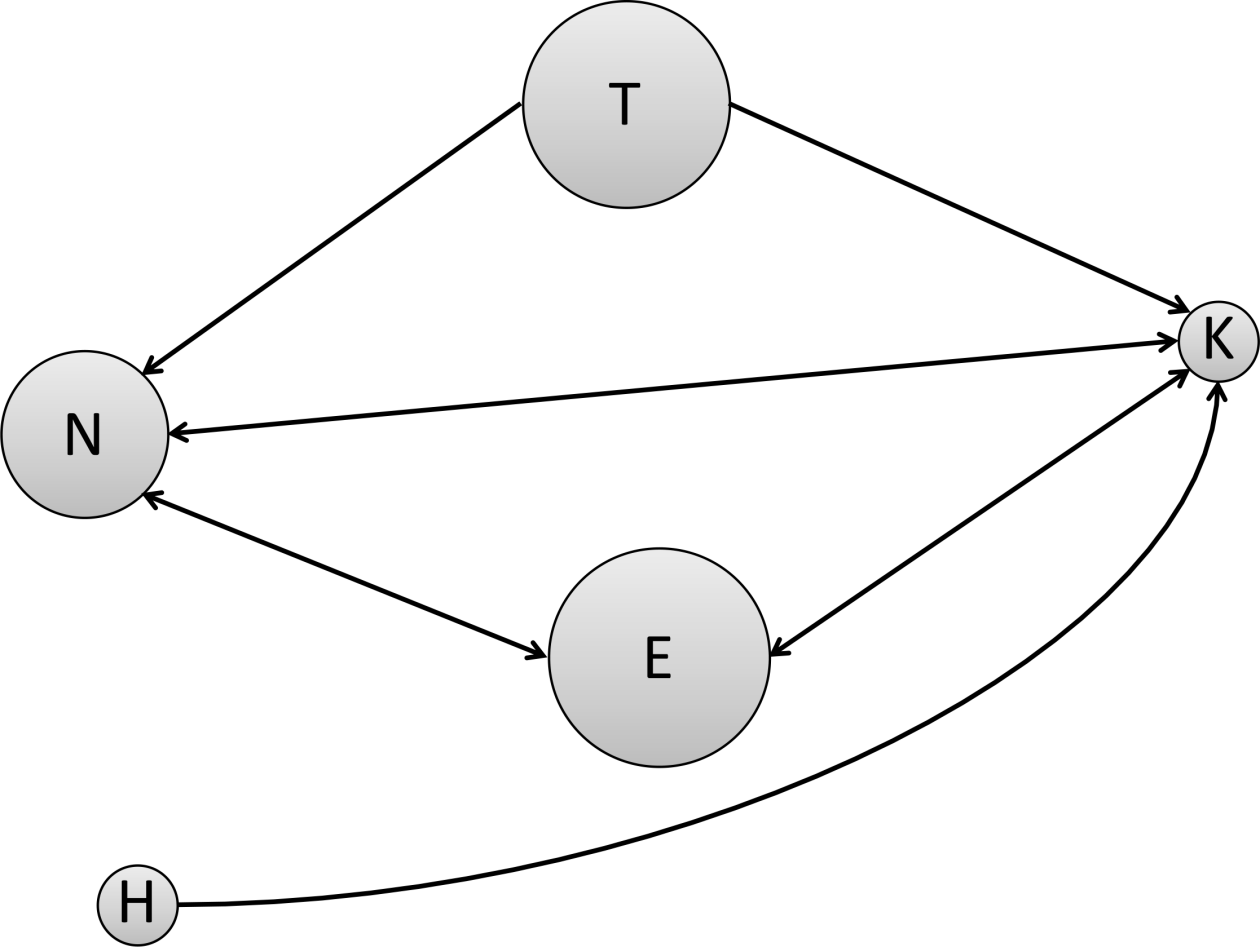
Estimated population size and migrations between populations. Long-term gene flow and population sizes estimated by MIGRATE-N are illustrated. The size of each circle reflects the effective population size of the indicated population. Arrows indicate migration between populations. The direction of the arrows indicates the direction of movement. Migration rates of *M* > 4 determined by MIGRATE-N are shown. Abbreviations: E, Erimo; H, Hakodate; K, Kitami; N, Nopporo; T, Teshio.

The migration rates calculated with BAYESASS+ program were uniformly very small, ranging from 0.0032 to 0.0050 for all population pairs (S4 Table). Although some of the migration rates estimated with the MIGRATE program were also low, rather high migration rates were estimated among the Erimo, Kitami, and Nopporo populations (Fig 4). The proportional index of effective population size (Θ) estimated by the MIGRATE program ranged from 0.5854 to 1.6275, with larger values in the Erimo, Teshio, and Nopporo populations (Θ = 1.6275, 95% credible interval 1.4971–1.7739; 1.5175, 1.4026–1.6823; and 1.2170, 1.1249–1.3191, respectively), and smaller values in the Hakodate and Kitami populations (Θ = 0.5854, 0.5436–0.6330; and 0.5928, 0.5516–0.6388, respectively).

## Discussion

We reconstructed a cladogram among local populations of Hokkaido salamander by using microsatellite markers. Although some parts of the phylogeny were consistent with the genealogy based on mitochondrial DNA [24], there were also inconsistencies. In our microsatellite analysis, only the Kitami and Teshio populations were clustered with high confidence, whereas the mitochondrial data analysis placed the Nopporo and Teshio populations into the same haplotype subgroup. The Ishikari-Yufutsu lowland frequently appears to act as a barrier in species distributions, including for taxa such as insects [46] and the Japanese brown bear (*Ursus arctos*) [47]. Nopporo is located at this lowland (Fig 1). During the last interglacial (130,000–60,000 years ago), the Ishikari-Yufutsu lowland was submerged, preventing migration [48]. Therefore, populations of the Hokkaido salamander probably became divided at that time. The Kitami and Teshio populations are clearly clustered in our STRUCTURE analysis for *K* = 2, and our microsatellite data are more informative than the mitochondrial data reported previously. Thus, we conclude that the genetic relationship indicated by our microsatellite data reflects the true phylogenetic relationships among all five populations.

By considering the two independent estimations of gene flow in combination, we concluded that migration occurred rather frequently in the distant past and much less frequently in the recent past. In particular, relatively high gene flow was indicated between the Nopporo and Kitami populations by the MIGRATE program, which estimates long-term migration rates, even though they are separated by high mountains (Kitami, Yubari, and Hidaka ranges). Landscape genetics studies of amphibians have shown that topography affects gene flow and that differences in altitude (such as mountains and valleys) specifically act as a barrier to gene flow between populations (e.g. [49–52]). Our result for these *H*. *retardatus* populations, however, implies that the varying topography did not act as barrier to gene flow, at least in some cases. Hynobidae species tolerate relatively low body temperatures and are abundant in mountainous areas of the Japanese archipelago [53]. The Hokkaido salamander in particular is well adapted to the cold weather on Hokkaido Island and easily moves from the lowlands into the mountains. Therefore, it may be able to migrate across different landscapes.

The estimated divergence time of *H*. *retardatus* is about 18–14 Ma [6], but the populations apparently did not diverge until about 0.72–0.32 Ma [24]. There are several possible explanations for this difference. Populations of the Hokkaido salamander experienced many environmental changes and two population bottlenecks [24]. The consequent demographic fluctuations, including population extinctions and recolonization, may have led to lineage sorting or to shallow divergences or both. Our estimated gene flow among populations may therefore reflect migration and colonization occurring in the distant past, but long after speciation.

## Conclusion

We clarified the population structure of the Hokkaido salamander and gene flow among populations. Populations of this species show distinct genetic differences and generally low gene flow. Thus, each population has different genetic features and evolved independently. These findings are applicable to the conservation management of this species. Because the populations are genetically clearly different, it is important to protect all of the local populations. In the future, a more detailed population structure and the evolutionary history of this species should be examined by using more comprehensive data, such as genome-wide polymorphic markers. The causal relationships between genetic background and the phenotypic differences among populations also need to be resolved.

## Supporting Information

S1 Table Allele frequency and sample size by population. (XLSX)

S2 Table Properties of 12 microsatellite loci in the Hokkaido salamander. (XLSX)

S3 Table Pairwise *F*_ST_ (below the diagonal) and *D*_A_ (above the diagonal) distances between populations. (XLSX)

S4 Table Estimated rates of gene flow between populations inferred by BAYESASS+ and MIGRATE-N. (XLSX)

S5 Table Genotyping data as genepop format. (XLSX)

## Abbreviations

HWE: Hardy-Weinberg equilibrium
LD: disequilibrium
MCMC: Markov Chain Monte Carlo
Ma: million years ago

## References

[1] Steinfartz S, Veith M, Tautz D (2000) Mitochondrial sequence analysis of *Salamandra* taxa suggests old splits of major lineages and postglacial recolonizations of Central Europe from distinct source populations of *Salamandra salamandra*. Mol Ecol 9: 397–410. 10736043 10.1046/j.1365-294x.2000.00870.x

[2] Donovan MF, Semlitsch RD, Routman EJ (2000) Biogeography of the southeastern United States: a comparison of salamander phylogeographic studies. Evolution 54: 1449–1456. 11005312 10.1554/0014-3820(2000)054[1449:botsus]2.0.co;2

[3] Zhang P, Chen Y, Zhou H, Liu Y, Wang X, Papenfuss T, et al (2006) Phylogeny, evolution, and biogeography of Asiatic Salamanders (Hynobiidae). Proc Natl Acad Sci U S A 103: 7360–7365. 16648252 10.1073/pnas.0602325103 PMC1464346

[4] Zheng Y, Peng R, Kuro-o M, Zeng X (2011) Exploring patterns and extent of bias in estimating divergence time from mitochondrial DNA sequence data in a particular lineage: a case study of salamanders (order Caudata). Mol Biol Evol 28: 2521–2535. 10.1093/molbev/msr072 21422243

[5] Weisrock DW, Macey JR, Matsui M, Mulcahy DG, Papenfuss TJ (2013) Molecular phylogenetic reconstruction of the endemic Asian salamander family Hynobiidae (Amphibia, Caudata). Zootaxa 3626: 77–93. 26176127 10.11646/zootaxa.3626.1.3

[6] Chen MY, Mao RL, Liang D, Kuro-o M, Zeng XM, Zhang P (2014) A reinvestigation of phylogeny and divergence times of Hynobiidae (Amphibia, Caudata) based on 29 nuclear genes. Mol Phylogenet Evol 83: 1–6. 10.1016/j.ympev.2014.10.010 25462999

[7] Nishikawa K, Matsui M, Tanabe S, Sato S (2001) Geographic enzyme variation in a Japanese salamander, *Hynobius boulengeri* Thompson (Amphibia: Caudata). Herpetologica 57: 281–294.

[8] Matsui M, Misawa Y, Nishikawa K, Tanabe S (2000) Allozymic variation of *Hynobius kimurae* Dunn (Amphibia, Caudata). Comp Biochem Physiol B 125: 115–125. 10840647 10.1016/s0305-0491(99)00154-6

[9] Tominaga A, Matsui M, Nishikawa K, Sato S (2003) Occurrence of two types of *Hynobius naevius* in northern Kyushu, Japan (Amphibia: Urodela). Zool Sci 20: 1467–1476. 14709811 10.2108/zsj.20.1467

[10] Yamane A, Nishida S (2010) Fine-scale spatial genetic structure and genetic diversity among clouded salamander (*Hynobius nebulosus*) populations. Curr Herpetol 29: 78–90.

[11] Matsui M, Nishikawa K, Tanabe S, Misawa Y (2001) Systematic status of *Hynobius tokyoensis* (Amphibia: Urodela) from Aichi Prefecture, Japan: a biochemical survey. Comp Biochem Physiol B 130: 181–189. 11544088 10.1016/s1096-4959(01)00424-9

[12] Matsui M, Tominaga A, Hayashi T, Misawa Y, Tanabe S (2007) Phylogenetic relationships and phylogeography of *Hynobius tokyoensis* (Amphibia: Caudata) using complete sequences of cytochrome b and control region genes of mitochondrial DNA. Mol Phylogenet Evol 44: 204–216. 17254807 10.1016/j.ympev.2006.11.031

[13] Sakamoto M, Tominaga A, Matsui M (2009) Phylogeography of *Hynobius yatsui* (Amphibia: Caudata) in Kyushu, Japan. Zool Sci 26: 35–47. 10.2108/zsj.26.35 19267610

[14] Sugawara H, Nagano M, Sueyoshi T, Hayashi F (2015) Local genetic differentiation and diversity of the Oita salamander (*Hynobius dunni*) in Kyushu revealed by mitochondrial and microsatellite DNA analyses. Curr Herpetol 34: 1–11.

[15] Sasaki M (1924) On a Japanese salamander, in Lake Kuttarush, which propagates like the axolotl. J Coll Agr Hokkaido Imp Univ 15: 1–36.

[16] Sakata N, Tamori Y, Wakahara M (2005) P450 aromatase expression in the temperature- sensitive sexual differentiation of salamander (*Hynobius retardatus*) gonads. Int J Dev Biol 49: 417–425. 15968587 10.1387/ijdb.041916ns

[17] Wakahara M (1995) Cannibalism and resulting dimorphism in larvae of a salamander *Hynobius retardatus*, inhabiting Hokkaido, Japan. Zool Sci 12: 467–473.

[18] Wakahara M (1997) Kin recognition among intact and blinded, mixed sibling larvae of a cannibalistic salamander *Hynobius retardatus*. Zool Sci 14: 893–899.

[19] Michimae H, Wakahara M (2002) A tadpole-induced polyphenism in the salamander *Hynobius retardatus*. Evolution 56: 2029–2038. 12449490 10.1111/j.0014-3820.2002.tb00129.x

[20] Iwami T, Kishida O, Nishimura K (2007) Direct and indirect induction of a compensatory phenotype that alleviates the costs of an inducible defense. PLoS One 2: e1084 17971850 10.1371/journal.pone.0001084 PMC2040518

[21] Matsunami M, Kitano J, Kishida O, Michimae H, Miura T, Nishimura K (2015) Transcriptome analysis of predator- and prey-induced phenotypic plasticity in the Hokkaido salamander (*Hynobius retardatus*). Mol Ecol 24: 3064–3076. 10.1111/mec.13228 25943778

[22] Michimae H, Nishimura K, Tamori Y, Wakahara M (2009) Maternal effects on phenotypic plasticity in larvae of the salamander *Hynobius retardatus*. Oecologia 160: 601–608. 10.1007/s00442-009-1319-8 19352721

[23] Matsui M, Sato T, Tanabe S (1992) Local population differentiation in *Hynobius retardatus* from Hokkaido: an electrophoretic analysis (Caudata: Hynobiidae). Zool Sci 9: 193–198.

[24] Azuma N, Hangui J, Wakahara M, Michimae H (2013) Population structure of the salamander *Hynobius retardatus* inferred from a partial sequence of the mitochondrial DNA control region. Zool Sci 30: 7–14. 10.2108/zsj.30.7 23317360

[25] Ohshima K (1990) The history of straits around the Japanese islands in the Late-Quaternary. The Quat Res 29: 193–208 [in Japanese with an English abstract].

[26] Kawai K, Hailer F, de Guia AP, Ichikawa H, Saitoh T (2013) Refugia in glacial ages led to the current discontinuous distribution patterns of the dark red-backed vole *Myodes rex* on Hokkaido, Japan. Zool Sci 30: 642–650. 10.2108/zsj.30.642 23915157

[27] Hirata D, Mano T, Abramov AV, Baryshnikov GF, Kosintsev PA, Vorobiev AA, et al (2013) Molecular phylogeography of the brown bear (*Ursus arctos*) in Northeastern Asia based on analyses of complete mitochondrial DNA sequences. Mol Biol Evol 30: 1644–1652. 10.1093/molbev/mst077 23619144

[28] Suzuki H, Yasuda SP, Sakaizumi M, Wakana S, Motokawa M, Tsuchiya K (2004) Differential geographic patterns of mitochondrial DNA variation in two sympatric species of Japanese wood mice, *Apodemus speciosus* and *A*. *argenteus*. Genes Genet Syst 79: 165–176. 15329497 10.1266/ggs.79.165

[29] Matsunami M, Igawa T, Nozawa M, Michimae H, Miura T, Nishimura K (2015) Development and characterization of 12 microsatellite markers for the Hokkaido salamander (*Hynobius retardatus*). Curr Herpetol 34: 177–181.

[30] Peakall R, Smouse PE (2012) GenAlEx 6.5: genetic analysis in Excel. Population genetic software for teaching and research—an update. Bioinformatics 28: 2537–2539. 22820204 10.1093/bioinformatics/bts460 PMC3463245

[31] Raymond M, Rousset F (1995) GENEPOP (version 1.2): population genetics software for exact tests and ecumenicism. J Hered 86: 248–249.

[32] Rousset F (2008) Genepop'007: a complete reimplementation of the Genepop software for Windows and Linux. Mol Ecol Resour 8: 103–106. 10.1111/j.1471-8286.2007.01931.x 21585727

[33] Chapuis MP, Estoup A (2007) Microsatellite null alleles and estimation of population differentiation. Mol Biol Evol 24: 621–31. 17150975 10.1093/molbev/msl191

[34] Putman AI, Carbone I (2014) Challenges in analysis and interpretation of microsatellite data for population genetic studies. Ecol Evol 4: 4399–428. 10.1002/ece3.1305 25540699 PMC4267876

[35] Van Oosterhout C, Hutchinson WF, Wills DPM, Shipley P (2004) Micro-Checker: software for identifying and correcting genotyping errors in microsatellite data. Mol Ecol Notes 4: 535–538.

[36] Nei M, Tajima F, Tateno Y (1983) Accuracy of estimated phylogenetic trees from molecular data. II. Gene frequency data. J Mol Evol 19: 153–170. 6571220 10.1007/BF02300753

[37] Takezaki N, Nei M, Tamura K (2010) POPTREE2: Software for constructing population trees from allele frequency data and computing other population statistics with Windows interface. Mol Biol Evol 27: 747–752. 10.1093/molbev/msp312 20022889 PMC2877541

[38] Hubisz MJ, Falush D, Stephens M, Pritchard JK (2009) Inferring weak population structure with the assistance of sample group information. Mol Ecol Resour 9: 1322–1332. 10.1111/j.1755-0998.2009.02591.x 21564903 PMC3518025

[39] Evanno G, Regnaut S, Goudet J (2005) Detecting the number of clusters of individuals using the software STRUCTURE: a simulation study. Mol Ecol 14: 2611–2620. 15969739 10.1111/j.1365-294X.2005.02553.x

[40] Earl DA, vonHoldt BM (2012) STRUCTURE HARVESTER: a website and program for visualizing STRUCTURE output and implementing the Evanno method. Conserv Genet Res 4: 359–361.

[41] Jakobsson M, Rosenberg NA (2007) CLUMPP: a cluster matching and permutation program for dealing with label switching and multimodality in analysis of population structure. Bioinformatics 23: 1801–1806. 17485429 10.1093/bioinformatics/btm233

[42] Rosenberg NA (2004) DISTRUCT: a program for the graphical display of population structure. Mol Ecol Notes 4: 137–138.

[43] Wilson GA, Rannala B (2003) Bayesian inference of recent migration rates using multilocus genotypes. Genetics 163: 1177–1191. 12663554 10.1093/genetics/163.3.1177 PMC1462502

[44] Beerli P, Palczewski M (2010) Unified framework to evaluate panmixia and migration direction among multiple sampling locations. Genetics 185: 313–326. 10.1534/genetics.109.112532 20176979 PMC2870966

[45] Pearse DE, Crandall KA (2004). Beyond *F*_ST_: analysis of population genetic data for conservation. Conserv Genet 5: 585–602.

[46] Kono H (1933) Importance of Hokkaido and Sapporo Lowland for butterfly fauna. Konchu 7: 86–88 (in Japanese).

[47] Matsuhashi T, Masuda R, Mano T, Yoshida MC (1999) Microevolution of the mitochondrial DNA control region in the Japanese brown bear (*Ursus arctos*) population. Mol Biol Evol 16: 676–684. 10335661 10.1093/oxfordjournals.molbev.a026150

[48] Japan Association for Quaternary Research (1987) Quaternary maps of Japan (plus explanatory text) University of Tokyo Press, Tokyo (in Japanese).

[49] Funk WC, Blouin MS, Corn PS, Maxell BA, Pilliod DS, Amish S, et al (2005) Population structure of Columbia spotted frogs (*Rana luteiventris*) is strongly affected by the landscape. Mol Ecol 14: 483–496. 15660939 10.1111/j.1365-294X.2005.02426.x

[50] Giordano AR, Ridenhour BJ, Storfer A (2007) The influence of altitude and topography on genetic structure in the long-toed salamander (*Ambystoma macrodactulym*). Mol Ecol 16: 1625–1637. 17402978 10.1111/j.1365-294X.2006.03223.x

[51] Murphy MA, Dezzani R, Pilliod DS, Storfer A (2010) Landscape genetics of high mountain frog metapopulations. Mol Ecol 19: 3634–3649. 10.1111/j.1365-294X.2010.04723.x 20723055

[52] Igawa T, Oumi S, Katsuren S, Sumida M (2013) Population structure and landscape genetics of two endangered frog species of genus *Odorrana*: different scenarios on two islands. Heredity (Edinb) 110: 46–56. 22990312 10.1038/hdy.2012.59 PMC3522236

[53] Matsui M (1996) Natural history of the amphibia University of Tokyo Press, Tokyo (in Japanese).

